# A Low-Profile Ultrawideband Antenna Based on Flexible Graphite Films for On-Body Wearable Applications

**DOI:** 10.3390/ma14164526

**Published:** 2021-08-12

**Authors:** Wenhua Li, Haoran Zu, Jinjin Liu, Bian Wu

**Affiliations:** National Key Laboratory of Antennas and Microwave Technology, Shaanxi Joint Key Laboratory of Graphene, Xidian University, Xi’an 710071, China; whli_2@stu.xidian.edu.cn (W.L.); zuhr@foxmail.com (H.Z.); jinjinliu@stu.xidian.edu.cn (J.L.)

**Keywords:** antenna, ultrawideband, flexible graphite films (FGF), low profile, omnidirectional, wearable application

## Abstract

This paper presents a low-profile ultrawideband antenna for on-body wearable applications. The proposed antenna is based on highly conductive flexible graphite films (FGF) and polyimide (PI) substrate, which exhibits good benefits such as flexibility, light weight and corrosion resistance compared with traditional materials. By introducing flaring ground and an arrow-shaped slot, better impedance matching is achieved. The wearable antenna achieves a bandwidth of 122% from 0.34 GHz to 1.4 GHz, with a reflection coefficient of less than −10 dB, while exhibiting an omnidirectional pattern in the horizontal plane. To validate the proposed design, the wearable antenna with a profile of ~0.1 mm was fabricated and measured. The measured results are in good agreement with simulated ones, which indicates a suitable candidate for on-body wearable devices.

## 1. Introduction

Wireless Body Area Networks (WBAN) have been widely applied in many aspects of novel wireless communication systems, e.g., health monitoring, military, and entertainment [1,2,3,4,5]. As a vital component in these systems, the wearable antenna is key to receiving the data from sensors while sending electric signals to the data register or base station for wireless wearable communication on or off-body channel communications (BCC) [6]. Ultrawideband technology is an attractive method for improved communications including wireless on-body networks. Various ultrawideband antennas have been reported such as Vivaldi form [7,8], microstrip form [9,10] and monopole form [11,12]. In these cases of single-layer antennas, it usually has a low profile, which shows great potential for wearable applications. Recently, wearable antennas have attracted much attention and have been rapidly developed [13,14,15,16,17]. However, there are several development bottlenecks for the design approach and performance requirements. The close vicinity of a lossy human body results in a reduction in antenna efficiency due to its high power consumption. Besides, the performance of a wearable antenna is sensitive to the different positions on the human body and different human bodies, such as men and women [18,19]. Simultaneously, the exposure of the human body to the electromagnetic field must be considered for safety. Therefore, a wearable antenna with a low specific absorption rate (SAR) is highly desired [20,21]. More importantly, for practical implementation, it is critical to design a flexible antenna that is bendable when worn by the user although the bending effects degrade the performance of antennas compared to their flat condition [22]. Prior research has reported lots of wearable button antennas with relatively small sizes, which can be easily mounted and make it unnecessary to fabricate using flexible materials [23,24,25]. However, flexible materials are in great demand when a wearable antenna operates at the lower frequencies. It can be noted that few of the proposed wearable antennas work at frequencies lower than 1 GHz where some of the frequency ranges are used for Medical Implant Communications Service (MICS, 402–405 MHz) and Wireless Medical Telemetry Services (WMTS, 608–614 MHz and 1395–1400 MHz) according to the IEEE 802.15.6 standard [26]. This is generally due to the bigger size of antennas working in the sub-GHz region. Several types of wearable antennas have been proposed using various materials in the past. In [27], a wearable microstrip antenna realized on a paper substrate based on conductive nanoink is presented. This compact structure operates in a sub-GHz band for wireless sensor networks. However, the antenna has narrow working bandwidth and large dimensions. In [28], a wideband and semi-flexible antenna is designed for wearable applications. The bandwidth is enhanced by using a hook-shaped stub resonator with the ground plane. However, a thick RT/duroid 5880 is used as a substrate, which makes it difficult to bend when worn on the body. In [29], Fang et al. propose a low-profile UWB antenna using graphene-assembled films (GAF). The highly conductive GAF and flexible ceramic substrate are adopted to ensure the flexibility and robustness of the antenna. However, the overall dimensions of the antenna seem to be large, which limits its applications in wearable devices. In [30], a flexible, body-worn fabric patch antenna based on conductive polymers (CPs) is firstly proposed without the use of metal. The antenna shows favorable flexibility and non-varying resonant frequency under various deformations. However, the fractional bandwidth of the antenna is only 15% at the center frequency of 2.35 GHz. Over the past decades, substantial flexible wearable antennas such as textile-based antennas combined with metallic thread have been developed [25,31]. Undoubtedly, these antennas have superiorities such as light weight and high flexibility while maintaining fabrication simplicity. Nevertheless, all these flexible structures are limited for practical wearable applications because the antenna performance is sensitive to temperature, humidity, and mechanical deformation, etc.

Recently, great attention has been paid to the flexible graphite films (FGF), which has been considered as a promising candidate for flexible wearable devices. These advanced carbon material based films exhibit unique properties including high conductivity, flexural endurance, light weight and corrosion resistance, which are studied in previous works [32,33].

In this paper, we propose a novel flexible ultrawideband antenna for on-body wearable applications. As far as we know, the FGF is firstly adopted to replace traditional metallic materials in wearable antenna design for the sub-GHz region, which shows great superiorities, especially in flexibility, mechanical stability and light weight. The simulated and measured results prove that the proposed antenna achieves an omnidirectional vertical polarized pattern and impedance bandwidth from 0.34 GHz to 1.4 GHz, which operates within the Ultra High Frequency (UHF) band (300–3000 MHz) for military applications [4] and the MICS and WMTS bands for medical applications [26]. Meanwhile, by adopting flexible polyimide (PI) substrate and coplanar waveguide (CPW)-fed method, the graphite-based wearable antenna has realized a low profile of ~0.1 mm, which is suitable for wearable devices.

## 2. Materials and Antenna Design

### 2.1. Characterization of Materials

**Figure 1:**
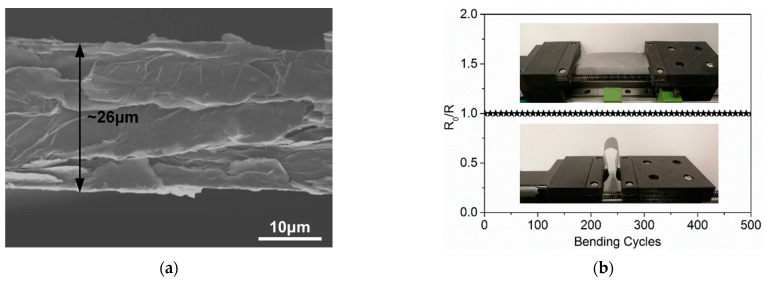
(**a**) Cross-section SEM image of the FGF. (**b**) Experiment of the mechanical reliability of the FGF.

In the field of wearable antenna design, various conductive materials are adopted to act as radiation structures such as conductive fabrics [9], copper [13], and textile-based materials [25,31], and so on. However, metallic materials exhibit unsatisfactory flexibility, while textile-based conductive materials show bad performance when exposed to humid circumstances. Compared with mentioned traditional conductive materials, the FGF shows excellent superiorities in light weight, favorable flexibility and corrosion resistance [32,33]. The FGF is prepared in the following three steps: vacuumed heating, firing under argon atmosphere, and rolling process, which is described in detail in [33]. The FGF we used in this paper comes from the Hubei Engineering Research Center of RF-Microwave Technology and Application in the Wuhan University of Technology. As shown in Figure 1, we describe the characteristic of the FGF in our previous work [32]. Figure 1a illustrates the cross-section scanning electron microscopy (SEM) image of the FGF sample, which shows the thickness of the FGF is ~26 μm, and the interrelating electrical conductivity is 1.1 × 10^6^ S/m, close to the traditional metallic like copper (1.3 × 10^7^ S/m). More importantly, the FGF is much lighter than the copper because the density of the FGF is 1.8 g/cm^3^, which is around a fifth of the copper density of 8.8 g/cm^3^ [33]. The experiment of mechanical reliability of the FGF is demonstrated in Figure 1b. It can be seen that the FGF maintains its unchanged resistivity after 500 times bending, which proves the FGF has good flexibility and mechanical stability.

In addition, the textile fabric is a porous, anisotropic and compressible material, whose structures and electromagnetic properties can be affected severely by the surroundings [34], thus flexible polyimide (PI) substrate is preferred to textile material. In this work, we choose PI as the substrate with a thickness of 0.1 mm, a dielectric constant of 3.5 and a loss tangent of 0.0027 (purchased from DuPont company of America).

### 2.2. Antenna Design

**Figure 2:**
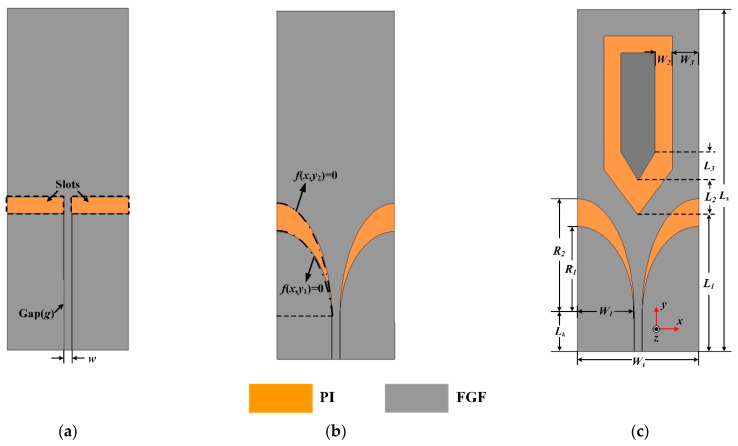
Proposed antenna design. (**a**) Ant-1: traditional monopole antenna. (**b**) Ant-2: modified monopole antenna. (**c**) Ant-3: ultrawideband wearable antenna.

Generally speaking, Microstrip antenna has a high quality factor (Q), which leads to a narrow bandwidth [35]. Compared with microstrip antenna, planar monopole antenna (PMA) is a common antenna that is widely utilized in wearable devices due to its simple structure, low profile and easy integration with the human body. The design progress of the proposed ultrawideband wearable antenna is exhibited in Figure 2. The initial antenna geometry is based on a typical planar monopole antenna. As depicted in Figure 2a, the prototype consists of a monopole patch and ground designed above the PI substrate. Both monopole patch and ground adopt the FGF for desired requirements. The proposed antenna is excited by a 50-ohm coplanar waveguide (CPW) for impedance matching, in which *w* and *g* are the widths of the central conductor and the gap between the central conductor and the ground, respectively.

**Figure 3:**
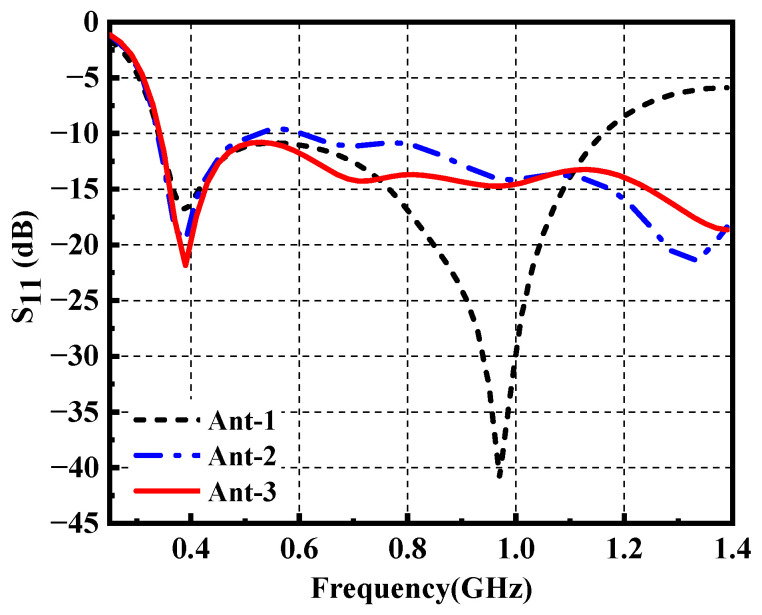
The reflection coefficient of the proposed antenna.

**Figure 4:**
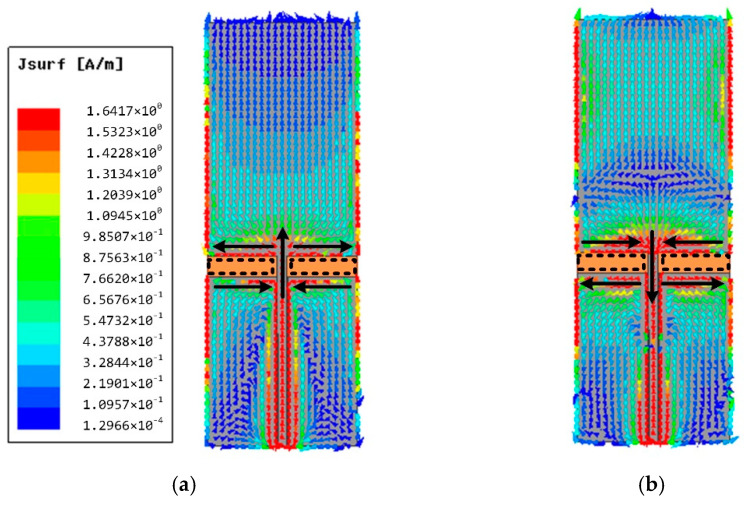
The surface current distribution of Ant-1. (**a**) 0.39 GHz. (**b**) 0.97 GHz.

All the simulations of the antenna are based on the ANSYS high-frequency structure simulator (HFSS). The simulated reflection coefficient S_11_ of the proposed antenna is shown in Figure 3. The traditional monopole antenna (Ant-1) has realized a bandwidth of 109% from 0.34–1.16 GHz (i.e., S_11_ < −10 dB), in which two resonances are observed at 0.39 GHz and 0.97 GHz. It is worth mentioning that the original antenna has realized the ultrawideband feature. The distribution plots of surface current at resonant frequencies above are given in Figure 4. It can be seen that Ant-1 operates in half-wavelength mode at 0.39 GHz and full-wavelength mode at 0.97 GHz, respectively. The currents of both sides of the slot show opposite directions, indicating the current distribution similar to a slot antenna. Therefore, the antenna in this design can be equivalent to a monopole antenna combined with two slot antennas.

To achieve a broader operating bandwidth, two rectangle slots are replaced with tapered slots by adopting flaring ground. As depicted in Figure 2b, the black dashed-dotted line corresponds to the outline of the flaring ground that satisfies the elliptical equation:

(1) $$\frac{x^{2}}{W_{1}^{2}}+\frac{y_{1}^{2}}{R_{1}^{2}}-1=0$$

(2) $$\frac{x^{2}}{W_{1}^{2}}+\frac{y_{2}^{2}}{R_{2}^{2}}-1=0$$

where *W*_1_ and *R*_1_ (*R*_2_) represent the minor-axis and major-axis radii of the elliptical patch, respectively. It can be seen from Figure 3 that the modified monopole antenna (Ant-2) has a broader bandwidth above 1.16 GHz compared with Ant-1. However, we can also observe that the reflection coefficient does not reach −10 dB around 0.55 GHz, which leads to performance degradation of the antenna.

**Figure 5:**
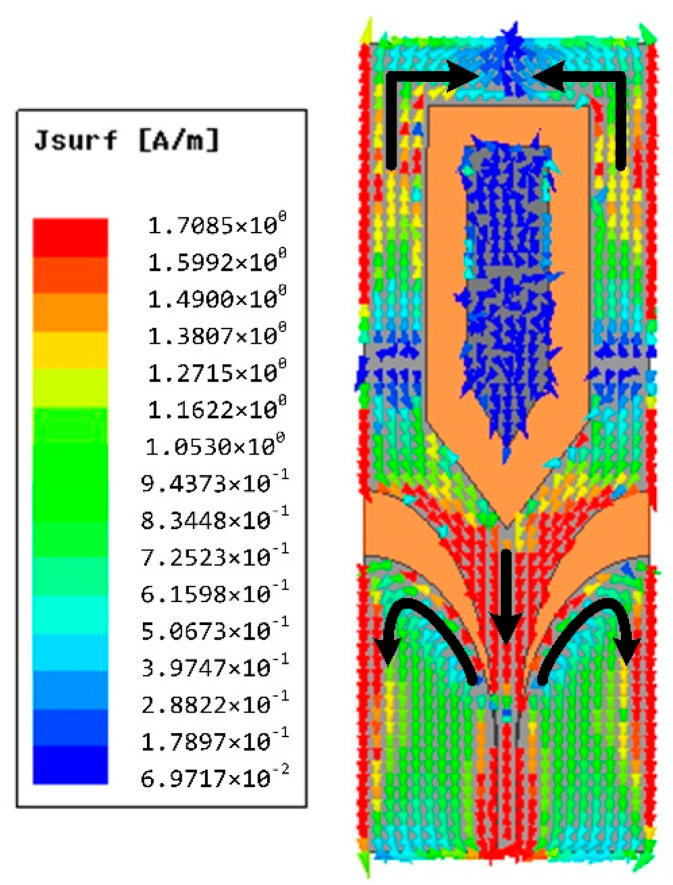
The surface current distribution of Ant-3 at 0.97 GHz.

**Figure 6:**
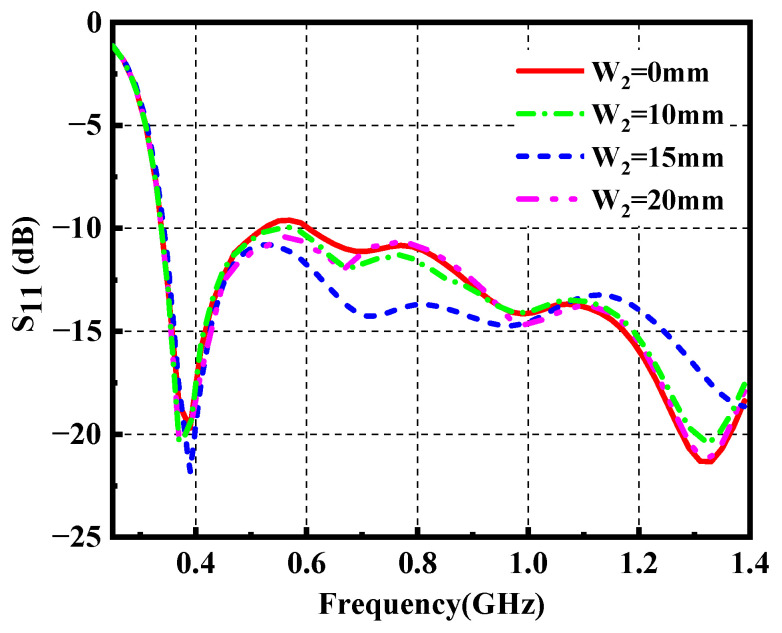
Simulated S_11_ of the Ant-3 with the variation of the *W*_2_.

Here a solution is presented by inserting an arrow-shaped slot structure on the radiation patch. Note that the flaring ground and the arrow-shaped slot are adopted for the wideband impedance matching. The simulated surface current distribution of the proposed antenna (Ant-3) is plotted in Figure 5. The surface current of Ant-3 increases around the arrow-shaped slot at 0.97 GHz compared with the one obtained of Ant-1, which helps to reduce S_11_ for the fractional bandwidth from 109% to 122%. The variation of S_11_ with *W*_2_ is shown in Figure 6. The arrow-shaped slot generates a capacitance effect, which helps to improve the impedance bandwidth and realize antenna miniaturization. Moreover, the current path of the Ant-3 becomes longer when *W*_2_ increases. However, when *W*_2_ is too large, the capacitance effect becomes weaker, which provides few contributions to impedance matching. Finally, we select the optimal value of 15 mm. Eventually, we obtain a flexible ultrawideband monopole antenna. Figure 2c depicts the geometry of the final ultrawideband wearable antenna (Ant-3). As shown in Figure 3, a comparison with the S_11_ obtained from the proposed antenna exhibits that the operating bandwidth of Ant-3 has been effectively improved with the fractional bandwidth of 122%. The optimized geometrical parameters of the proposed antenna are listed in Table 1.

**Figure 7:**
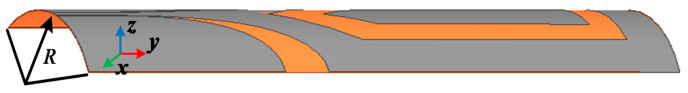
The wearable antenna prototype bent along the *y*-axis.

**Figure 8:**
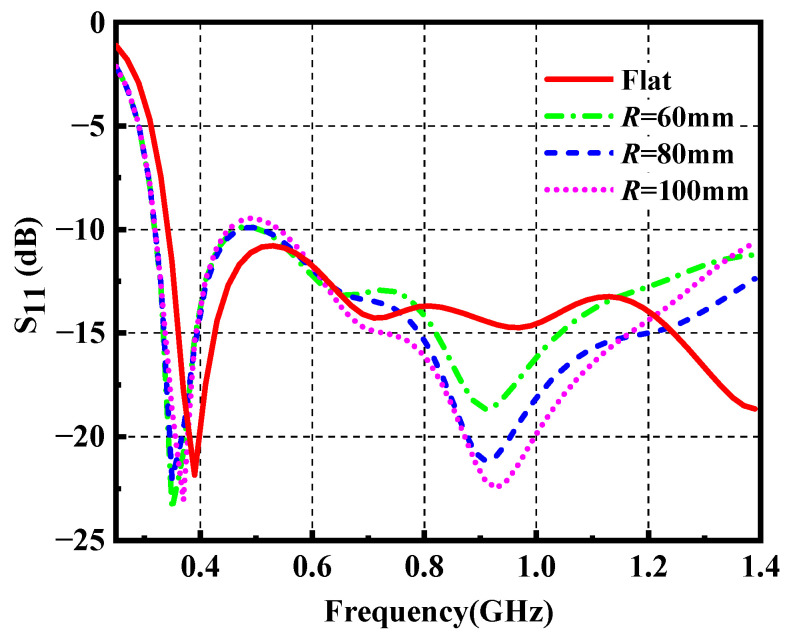
Reflection coefficient of the wearable antenna under flat and bending states.

There is no doubt that the bending effects of the wearable antenna degrade the performance compared with its flat condition, and the wearable antenna performance will be also affected by unavoidable deformation even though the antenna is designed for a certain bending radius. In this design, the proposed ultrawideband wearable antenna is conformally integrated over a cylindrical foam with a radius of *R*. By taking bending states of the body surface into account, we set the bending radii of *R* = 60 mm, 80 mm and 100 mm. As plotted in Figure 7, the cylindrical surface on which the antenna is bent is oriented along the *y*-axis. Figure 8 demonstrates the reflection coefficient of the wearable antenna at different *R*, which indicates a slight degradation of S_11_ around 0.5 GHz and a shift of resonant frequency to lower frequency as well. It proves that the impedance matching of the wearable antenna is well maintained compared with its flat condition.

**Figure 9:**
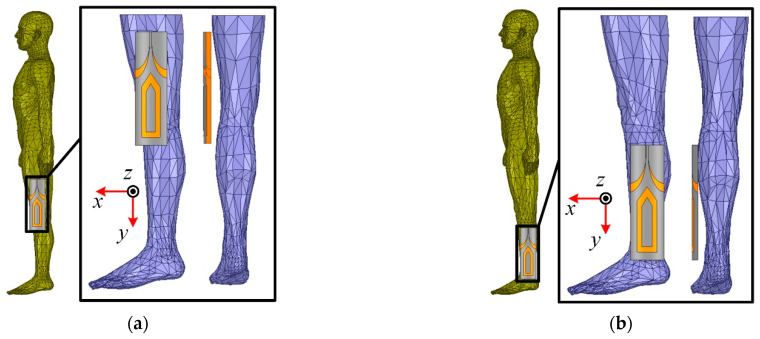
The wearable antenna loaded on the different positions of the voxel model. (**a**) Thigh area. (**b**) Shank area.

**Figure 10:**
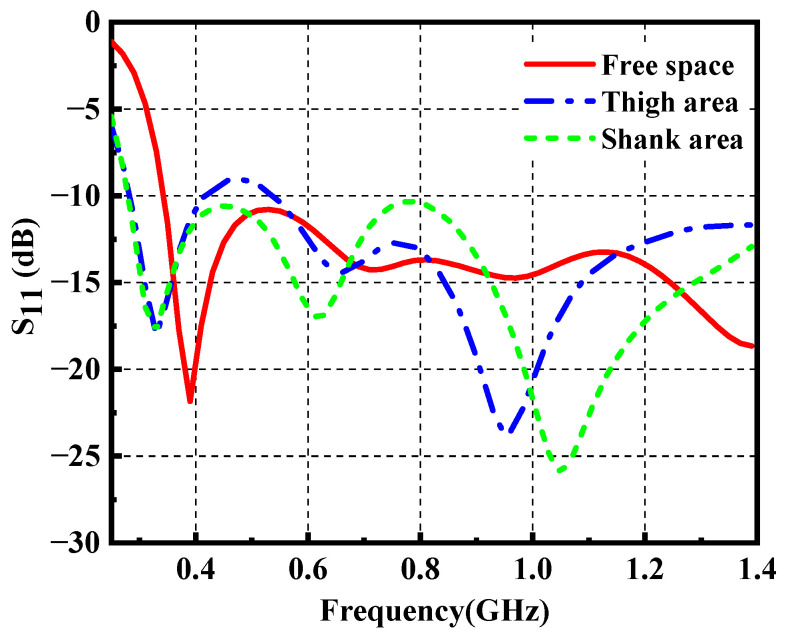
Reflection coefficient of the wearable antenna in free space, thigh area and shank area.

To study the impact of the loading positions of the proposed antenna, the antenna performance in free space, thigh area and shank area is simulated. As depicted in Figure 9, the three-dimensional voxel model of the male of ANSYS HFSS is adopted to mimic real scenarios. The wearable antenna is located near the thigh area and shank area (10 mm above body surface) due to its dimension. This is done by attaching the antenna onto a cylindrical foam, with a diameter of 100 mm and 80 mm (not shown in the photograph), respectively. Figure 10 demonstrates the simulated reflection coefficient in free space, thigh area and shank area, which plots the resonant frequency shift to the lower frequency. Meanwhile, impedance mismatch occurs around 0.5 GHz when the antenna is mounted near the thigh area. Both of them are due to the high dielectric constant of the human body.

**Figure 11:**
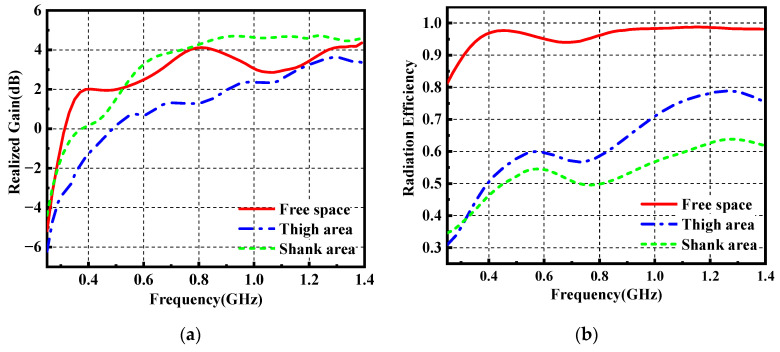
Realized gain (**a**) and radiation efficiency (**b**) of the wearable antenna in free space, thigh area and shank area.

**Figure 12:**
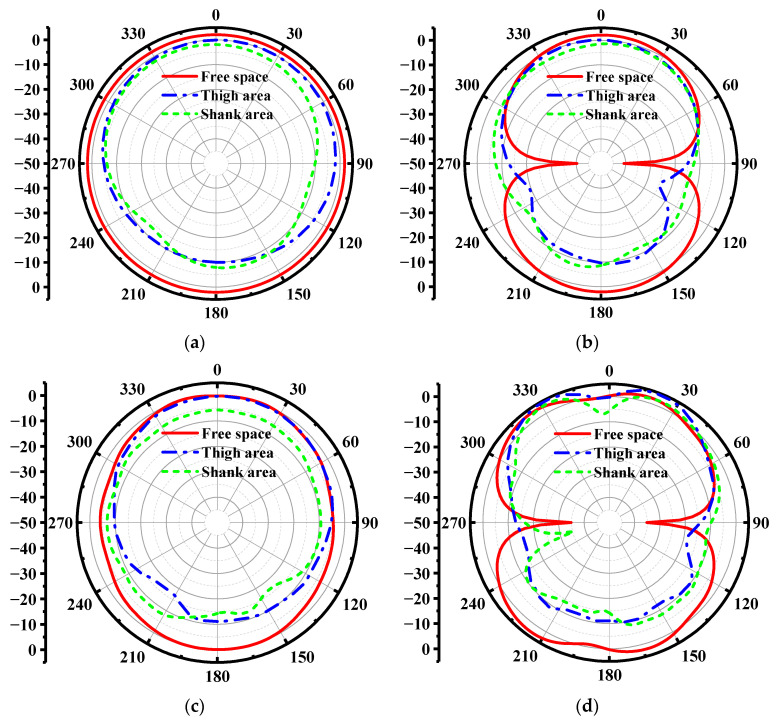
The radiation patterns of the wearable antenna in free space, thigh area and shank area. (**a**) 0.4 GHz in xoz plane. (**b**) 0.4 GHz in yoz plane. (**c**) 1.2 GHz in xoz plane. (**d**) 1.2 GHz in yoz plane.

The simulated realized gain and radiation efficiency are illustrated in Figure 11a,b, respectively. We can observe that the realized gain of the antenna is not decreased severely due to the electromagnetic reflection of the body. However, as shown in Figure 11b, the radiation efficiency of the wearable antenna reduces from 95% to 60% because of the proximity of the lossy human body, which limits the power transmission in free space. As displayed in Figure 12, the wearable antenna has an omnidirectional pattern in the xoz plane and 8-shape patterns in the yoz plane when loaded near the human body. Besides, the gain at 0° direction is higher than the gain at 180° direction, which is mainly caused by the absorption of the human body.

## 3. Measurement and Results

**Figure 13:**
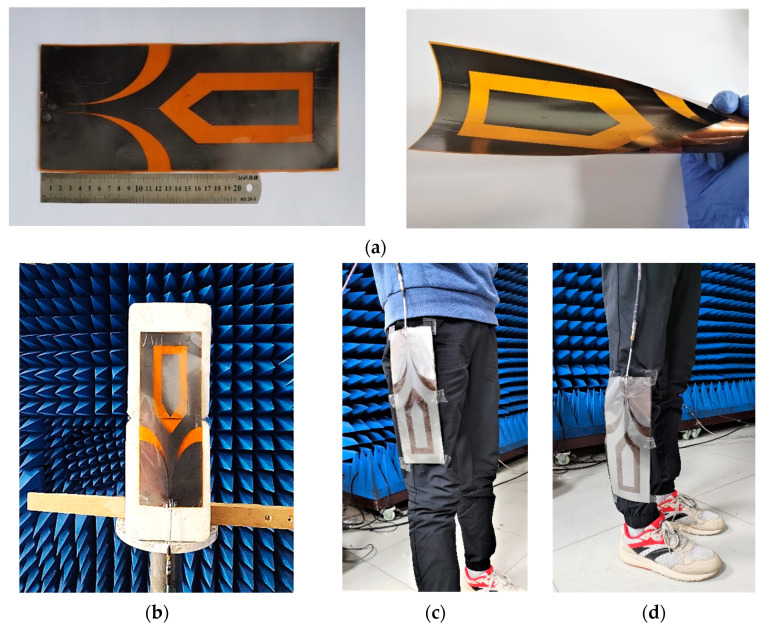
(**a**) Photographs of the fabricated wearable antenna. Experiment setup for different loading positions. (**b**) Free space. (**c**) Thigh area. (**d**) Shank area.

**Figure 14:**
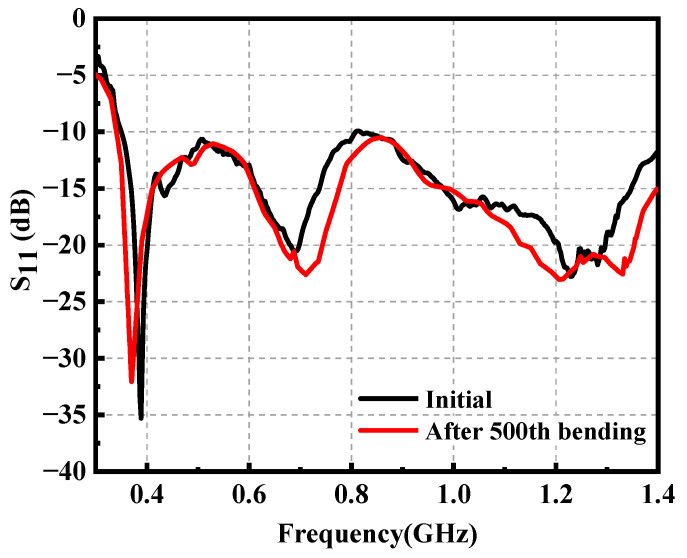
The reflection coefficient of the proposed antenna before and after bending.

**Figure 15:**
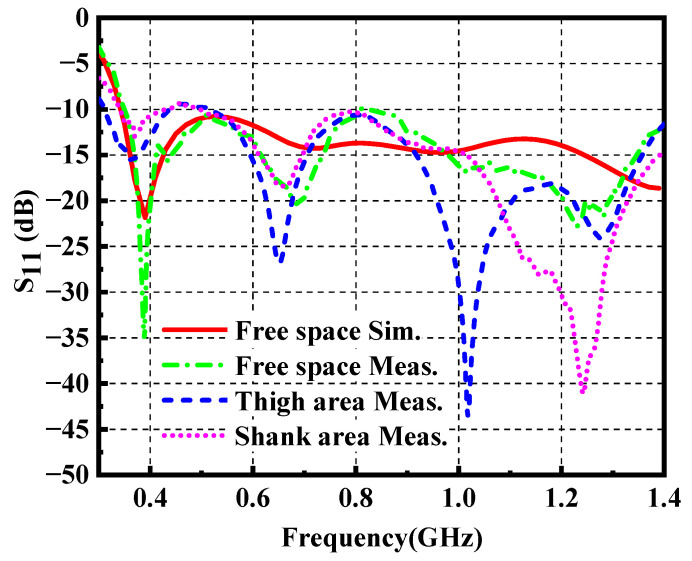
The simulated and measured reflection coefficient of the proposed antenna.

**Figure 16:**
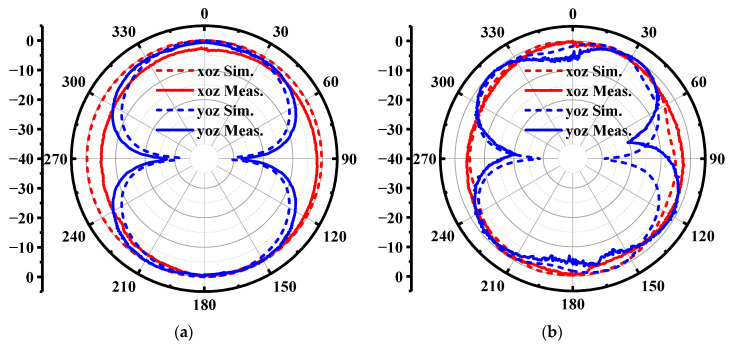
The simulated and measured radiation patterns of the wearable antenna in free space. (**a**) 0.4 GHz. (**b**) 1.2 GHz.

As shown in Figure 13, one prototype of the wearable antenna was fabricated and measured to validate the proposed antenna design. A LPKF laser engraver machine is used to outline the FGF-based monopole patch with desirable shapes on a PI substrate, which is described in detail in [33]. The wearable antenna and standard gain log periodic antenna were connected to the vector network analyzer (VNA). As presented in Figure 14, the bending effect to the antenna is negligible, which not only exhibits the great property of the material such as flexibility and mechanical stability, but also indicates the stable performance of the antenna under bending conditions. To prove the practicability of the wearable antenna, the fabricated antenna was attached to two positions including the thigh area and shank area. Figure 15 provides the measured reflection coefficient, which shows a good agreement with the simulated result, except for a slight frequency deviation in the high-frequency band. It may be caused by manufacturing errors and different dielectric constants corresponding to different positions. It is worth noting that the real human body increases the impedance matching bandwidth in the low-frequency band compared with numerical results, which indicates that most of the input power has been transmitted to the antenna. The simulated and measured radiation patterns at 0.4 GHz and 1.2 GHz are given in Figure 16, which has exhibited an omnidirectional pattern in the xoz plane as well as reached a good consistency, despite slight degradations in minor directions. Table 2 lists the comparison between the presented wearable antenna and other existing wide-band wearable antennas. It can be concluded that our work realizes ultrawideband characteristics in the sub-GHz region with the lowest profile. More importantly, the adoption of the FGF and PI substrate guarantees the favorable flexibility, mechanical stability and light weight of the wearable antenna.

## 4. Conclusions

A novel ultrawideband antenna using highly conductive flexible graphite films (FGF) for on-body wearable applications is proposed, fabricated and tested. The ultrawideband characteristic is realized by modifying the planar monopole antenna using the flaring ground and the arrow-shaped slot. In addition, to satisfy some special performance of the wearable antenna, we adopt the FGF as the conductive material, which shows a good candidate for wearable devices. To the best of our knowledge, it is the first time to adopt the FGF to replace traditional metallic materials in wearable antenna design within the sub-GHz region. The fabricated prototype has only a profile of 0.1 mm, making it applicable for conformal and wearable applications. The measured results have demonstrated that the FGF antenna operates from 0.34 GHz to 1.4 GHz with fractional bandwidth of 122% while providing an omnidirectional vertical polarized pattern in the xoz plane although it is bent and working in the proximity of the human body. The proposed antenna has many superiorities such as low profile, favorable flexibility, light weight, ultrawideband, and omnidirectional beam pattern, which may be suitable for an on-body communication system. This work has also demonstrated that the FGF has great potential in wearable antenna design.

## Figures and Tables

**Table 1:** Geometrical parameters of the proposed antenna.

Parameter	Value (mm)	Parameter	Value (mm)
*W_s_*	106	*L*_1_	120
*W*_1_	49.2	*L*_2_	30
*W*_2_	15	*L*_3_	25
*W*_3_	23	*L_k_*	35
*R*_1_	75	*L_s_*	300
*R*_2_	99.3	*h*	0.1
*w*	6.7	*g*	0.36

**Table 2:** Comparison of the proposed antenna with other wideband wearable antennas.

Ref.	Structure	Size	Profile (mm)	Bandwidth (%)	Frequency Range (GHz)	Material (Conductive/Dielectric)
[9]	Microstrip	1.86 $\lambda_{0}$ × 1.56 $\lambda_{0}$ (@6.9 GHz)	3.4	93	3.68–10.1	Conductive Fabrics/PDMS
[11]	Monopole	0.47 $\lambda_{0}$ × 0.33 $\lambda_{0}$ (@2.0 GHz)	~0.7	109	1.198–4.055	Copper Taffeta/Polyester
[12]	Monopole and Loop	0.29 $\lambda_{0}$ × 0.20 $\lambda_{0}$ (@4.0 GHz)	~1.1	148	2.15–14.75	NA./Rogers 6010
[27]	Monopole Above AMC	0.18 $\lambda_{0}$ × 0.18 $\lambda_{0}$ (@0.9 GHz)	~4.3	37	0.75–1.1	Conductive Nano-ink and Resin-coated Paper/Polycarbonate
[28]	Microstrip	0.20 $\lambda_{0}$ × 0.29 $\lambda_{0}$ (@2.4 GHz)	~0.8	59	1.62–3.0	NA./RT/duroid 5880
[29]	Monopole	0.64 $\lambda_{0}$ × 1.04 $\lambda_{0}$ (@6.0 GHz)	~0.3	67	4.0–8.0	Graphene-assembled Film/Ceramic
Our work	Monopole	0.87 $\lambda_{0}$ × 0.30 $\lambda_{0}$ (@0.87 GHz)	~0.1	122	0.34–1.4	FGF/PI

Where $\lambda_{0}$ is the free space wavelength corresponding to the center frequency.

## Data Availability

Not applicable.
